# Cancer and the Scientific Spirit

**Published:** 1916-03-18

**Authors:** Edwin Ash

**Affiliations:** Harley Street, W.


### THE EDITOR'S LETTER-BOX.

**Our Correspondents are reminded that brevity of style and conciseness of statements greatly facilitate early insertion. We cannot in any way be responsible for the opinions expressed by correspondents, who must give their name and address as a guarantee of good faith, but not necessarily for publication.**

### Cancer and the Scientific Spirit.

*To the Editor of* THE HOSPITAL.

SIR,—I have read the interesting article on the cancer problem (THE HOSPITAL, March 4, 1916) in relation to your excellent remarks on the scientific spirit contained in the same issue of your journal anent the review of Dr. P. Chalmers Mitchell's book on "Evolution and the War," and, having done so, am stimulated to send you the following observations.

Surely it is because the scientific spirit is not always followed by medical men that such cancer cures "within the profession" as those to which you refer have obtained reputable recognition, although they have one and all failed to make good, for he who approaches the cancer problem in an unscientific manner may readily tumble into one of many pitfalls that are spread to the unwary; indeed, the careless observer has never to go far before he comes across what appears to be evidence that some particular remedy has "cured" a case of cancer.

I think the following recent experience may serve as a useful illustration of these pitfalls. About seven months ago there came into my care a lady of middle age who was seriously ill from a condition diagnosed as carcinoma of the uterus by two surgeons whose opinion had been confirmed by a well-known gynæcologist on the staff of one of our largest teaching hospitals; the latter authority, indeed, expressed the opinion that the case was inoperable. The patient had also been attended for a time by a third doctor, who apparently found no reason to disagree with the diagnosis.

Up to this point, then, the patient had been seen by four London doctors, one of them a leading specialist, all of whom diagnosed the disease from which she was suffering as advanced cancer of the uterus. She was then placed in my care, and, after examining the patient, I also came to the conclusion that she was certainly suffering from advanced cancer, and accepted the surgical opinion that the growth was inoperable. A leading feature of the illness at that time was agonising pain referred to the back, and which could only be kept under by substantial doses of morphia injected hypodermically.

Now, Sir, doctors pride themselves on fighting their battles to the last ditch, and in this case, rather than throw up the sponge, I decided to give injections of the vaccine prepared from *Micrococcus neoformans*, which in the hands of Sir Almroth Wright and other workers is said to have retarded the progress of cancerous growths, and at times to have relieved pain and distress through antagonising secondary inflammatory conditions. To cut a long story short, the patient began to get better, and to-day (March 10, 1916), after six months in the nursing home where she was sent to die, has once more gone out into the world to take up her ordinary life with her friends and relatives, being, so far as her general health is concerned, apparently better than she has ever been for a long time. I ought to say that a mass which could be felt on palpating the abdomen seems to have disappeared.

Here, then, is a case which no less than five doctors were satisfied to consider as one of inoperable cancer, apparently "cured" by the injection of a vaccine. (Seven injections, representing about twenty million micrococci for each dose, were given during the six months.)

But let us examine the facts in the light of the scientific spirit, and it will quickly be seen that one has no right to claim this as a cure, for no scientific worker should assert that any diagnosis of cancer is incontrovertible, however numerous or distinguished the doctors who make it, unless one or other of the following conditions has been fulfilled:—

1. Confirmation by microscopical examination.
2. Such progress of the disease as by definite objective evidence leaves no doubt as to the nature of the underlying process.

In the example cited neither of these conditions has been fulfilled, and I bring it to your notice as an instance in which false deductions might readily be made by any medical man who permitted his keen interest and his delight at the successful issue of the case to obstruct his reasoning faculties.

It will be asked what, then, do I think of my case? I can only say that my data are insufficient for me to express an opinion. To clear things up as far as possible, it had been my intention to call in the distinguished gynæcologist who saw the patient some six months ago, but, to the regret of all who knew him, he himself was taken suddenly ill, and died only a few days ago. On the evidence it is impossible to say if the case was really one of cancer (or, if cancer, whether it has really "disappeared"), or if it was one of some simple condition that happened to react favourably. On the other hand, one can understand that enthusiastic persons might gamble on the unanimous opinion of the "five doctors" and claim that a miracle had been wrought.—I am, faithfully yours,

EDWIN ASH, M.D.

Harley Street, W., March 10, 1916.

